# Cohort-based analysis of paternal opioid use in relation to offspring’s BMI and plasma lipid profile

**DOI:** 10.1038/s41598-021-88781-9

**Published:** 2021-05-04

**Authors:** Zahra Jalali, Saeed Bahrampour, Parvin Khalili, Morteza Khademalhosseini, Ali Esmaeili Nadimi

**Affiliations:** 1 Non-Communicable Diseases Research Center, Rafsanjan University of Medical Sciences, Rafsanjan, Iran; 2 Department of Clinical Biochemistry, School of Medicine, Rafsanjan University of Medical Sciences, Rafsanjan, Iran; 3 Student Research Committee, Rafsanjan University of Medical Sciences, Rafsanjan, Iran; 4 Social Determinants of Health Research Centre, Rafsanjan University of Medical Sciences, Rafsanjan, Iran; 5 Department of Pathology, School of Medicine, Rafsanjan University of Medical Sciences, Rafsanjan, Iran; 6 Department of Cardiology, School of Medicine, Rafsanjani University of Medical Sciences, Rafsanjan, Iran

**Keywords:** Biochemistry, Diseases, Risk factors

## Abstract

A growing body of evidence suggests that opioid use may affect consumer’s offspring by second-hand passive smoke exposure, as well as by transgenerational impacts mediated by genetic and epigenetic alterations of paternal gametes. In human studies, these effects are limited to investigating the neural, behavioral and cognitive characteristics of offspring. Only animal studies have investigated the metabolic parameters influenced by passive opium smoke exposure. Here, we conducted population-based analyses aimed to estimate the association of paternal opioid consumption, started before or after child birth, with BMI status and plasma lipid profile of young adult offspring. The present study includes 840 parents-offspring trios (offspring aged 15–35, parents aged 35–70) who participated in the prospective Rafsanjan Cohort Study (RCS)—a city in the south-east of Iran—as one of the district areas of the PERSIAN cohort (Prospective Epidemiological Research Studies in IrAN). All procedures for interviews, anthropometric measurements and physical examinations, biological sample collection and laboratory tests for blood biochemical parameters were conducted according to the PERSIAN cohort protocol, and in the well-established RCS setting. Crude and adjusted multiple logistic regression analysis were conducted to assess the relationship of paternal regular opioid use with offspring’s BMI status, and plasma lipid factors. The prevalence of fathers who use opioids regularly among the studied trios was 42.8% (360/840). Our regression analyses demonstrated that paternal opioid use started pre-fatherhood is associated with 76% higher adjusted odds ratio (OR) of overweight/obesity in young offspring (adjusted OR 1.76 (95% CI 1.15–2.71)), adjusting for sex, age, parental BMIs, paternal smoking status and socioeconomic status index (WSI). This relationship persisted when fathers who used opioid by routes other than inhaling (oral) were excluded from logistic analysis (adjusted OR 1.73 (95% CI 1.12–2.68)). Interestingly, sex stratified analysis displayed a 201% increased odds ratio of overweight/obesity in sons of fathers who use opioid regularly, started after child birth (Adjusted OR 3.01 (95% CI 1.68–5.39), while no significant association was found in daughters (adjusted OR 0.74 (95% CI 0.35–1.54)). Additionally, increasing exposure–response relationships were observed between odds ratios of overweight/obesity and the number of years of paternal opioid use after birth (p-trend = 0.0008). Paternal regular opioid use started pre-fatherhood was associated with 54% lowered risk of underweight [adjusted OR 0.46 (95% CI 0.24–0.86)]. Finally, paternal opioid consumption started either before or after child birth did not show a significant association with the high level of the three parameters of plasma lipid factors (triglyceride, cholesterol and HDL-cholesterol) in offspring. Our results suggest that the environmental impacts of paternal regular opioid use may be sufficient to make an effect on male offspring metabolism independent of genetic and epigenetic impact on gametes.

## Introduction

Before COVID-19 pandemic, non-communicable diseases (NCDs) were considered as the leading global public health burden of the twenty-first century^1,2^, accounting for around 38 million death annually^3^. It was predicted that by 2025, NCDs will be the underlying cause of 70% of all death worldwide^4^. The prevalence of metabolic diseases and obesity is increasing around the world, specifically concerning among youth and children^5^. A growing body of evidence supports the notion that obesity and metabolic diseases are programmed early in life^6,7^; and prenatal conditions, as well as early postnatal life exposures can affect the risk of developing obesity and impaired metabolism in adulthood^8^.

The role of parental drug use on offspring weight and metabolic status are mainly addressed in studies which investigated the effects of maternal line cocaine exposure on offspring, assessing various effects including diabetes, obesity and hypertension in adulthood^9^. Opioid derivatives are one of the most commonly used illicit drugs worldwide. Parental opioid exposure studies remain limited to investigations on birth defects, preterm birth, fetal growth, withdrawal syndrome and other neonatal birth-time outcomes, as well as neural, behavioral and cognitive development. Therefore, late-onset lifetime metabolic complications of the offspring are not addressed yet^9–13^. Opioid route of consumption involves smoking specifically about heroin opium, and animal studies provide evidences that it may exert second hand/passive effects on cardiovascular indices and metabolic parameters such as plasma lipid profile^14^. In addition, opioids have been implicated in multi and transgenerational effects through epigenetic and genetic alterations of the germ-line; although these studies are limited to neural and behavioral aspects^15,16^, and no human reports of transgenerational metabolic effects of opioids has been released yet. Overall, little is known about the effects of parental opioid consumption (passive smoke/genetic/epigenetic) on late-onset complications such as diabetes, metabolic syndrome and obesity, warranting more investigation in this field. Here, we conducted a cohort-based population study, using data obtained for 840 parents-child trios in the prospective Rafsanjan Cohort Study (RCS). RCS is one of the 19 district areas of the PERSIAN cohort^17^, launched in 2014 and consists of four parallel studies: adults (aged 35–70)^18^, youth (aged 15–35), birth and copper-mine occupational cohorts. In the present study, the prevalence of regular opioid use by fathers was estimated to be around 42.8% among the 840 trios of parent–child in RCS. In the current study we measured the association of paternal regular opioid use started before or after child birth with the offspring’s BMI status, as well as plasma lipid parameters triglyceride, cholesterol and HDL.

## Materials and methods

### Subjects, study design and ethical considerations

In the present cross-sectional study, subjects were 840 young adults (aged 15–35) enrolled in youth Rafsanjan Cohort Study (RCS)^18^, whose parents have also participated in the adult RCS (aged 35–70) (840 trios of parents and offspring). The Persian Youth Cohort (PYC) is a part of the comprehensive PERSIAN (the Prospective Epidemiological Research Studies in IrAN) which started in 2014 and involves 9 thousands of 15–35 years old young adults in three cities of Iran including Rafsanjan city located in east-south region. Among the 3000 participants of Rafsanjan youth cohort participants, 840 individuals are included in the present study (because of the participation of both of their parents in the adult RCS^18^; and after exclusion of subjects with incomplete laboratory test results or questionnaires for the offspring or either parent).

All invitations, interviews, measurements and physical examinations were performed under the supervision of the Iranian Ministry of Health and Medical Education and the PERSIAN Cohort Central Committee, and in accordance with the PERSIAN cohort’s protocol^17^. In addition, all the protocols of the present study were approved by the Ethics Committee of Rafsanjan University of Medical Sciences (Ethical codes: IR.RUMS.REC.1398.043). Participants, who accepted invitations, entered the study voluntarily. Informed consent was obtained from every participant of RCS for the interview, physical examinations, bio-specimen collection and the use of the collected data for research. For participants younger than 18, informed consent was obtained from the parents on their behalf. All necessary measures were taken to ensure confidentiality of personal data of participants.

### DATA collection and measurements

All participants underwent comprehensive interviews, physical examinations by medical doctors, and biological sample donation. Interviews were performed by trained experts in a computer-assisted central server-connected fashion with standardized, validated questionnaires regarding demographic information, medical history, habits such as smoking and substance use, etc. Blood pressure and anthropometric measurements were performed by trained health professionals. Blood Biochemical parameters were measured using a CPALS analyzer (Coultronics, Margency, France) at the Central Laboratory in Rafsanjan Cohort center.

Opioid was considered as opium and opium-derivatives heroin, sukhteh and shireh^19,20^. RCS uses comprehensive and detailed questionnaires regarding substance use and smoking status including information on the age of commencement, duration of usage, type, amount and the frequency of substance use and the routes of administration. Opioids in this geographical region are used in two routes of smoking and oral consumption. The opioid derivatives reported among our participants were heroin, opium (Teriak), Sukhteh, and Shireh. Opium is prepared after air-drying the raw opium and consumed via pipe-smoking or orally^19,20^. Sukhteh is a black dry residue attached to the teriak pipe post-smoking and is consumed orally. Shireh is a refined product of opium which is a filtered product of boiling raw opium and Sukhteh in water consumed via pipe smoking^19,20^. In this study, parents who used opioid regularly were divided to those who started the habit from before child birth, or after, in order to distinguish if any potential epigenetic transgenerational impact may be involved. Regular opioid use was defined as opioid consumption for at least 1 day per week for at least 1 year continuously (every week of the year); and smokers were defined as individuals who reported daily smoking for at least 1 year continuously. We calculated BMI as the ratio of weight (kg) to squared height (m^2^). Participants were divided to four categories based on BMI (underweight, below 18.5; Normal or Healthy Weight 18.5–24.9, Overweight 25.0–29.9, Obese 30.0 and Above). In order to support sufficient power for statistical logistic analysis, we had to collapse the categories in to two categories, so that in analyzing odds ratio of overweight/obesity the two categories are participants with BMI ≥ 25 or BMI < 25; and for underweight, participants are divided to two categories with BMI < 18.5 or BMI ≥ 18.5). For analysis of serum lipid profile components, serum Tg ≥ 150 mg/dl, HDL-cholesterol < 40 mg/dl for men and < 50 for women and total cholesterol > 200 were considered groups with parameters of dyslipidemia. Parental hypertension, diabetes and obesity counts are based on self-reported information recorded by comprehensive medical questionnaires. All participants of adult and youth in RCS have been enrolled in the study providing valid ID and the familial relationships have been recorded accurately by attributing a specific PCID (personal code identification) which includes information on familial characteristics.

### Statistical analyses

The chi-square test was used to analyze categorical variables across youth; for continuous variables t-test was applied. In addition, in cases where more than 20% of the expected counts were less than 5, Fisher's Exact test was used. Bivariate and multiple logistic regression analyses were undertaken to assess the relationships between paternal opioid use started before and after child birth, and offspring BMI status and plasma lipid components (triglyceride, cholesterol and HDL). Potential confounding parameters were recognized based on subject matter knowledge and the relevant epidemiological literature. Separate models at bivariate level were run to estimate the strength of association of each variable with youth weight, hypertriglyceridemia, low HDL and hypercholesterolemia. Next, variables which displayed a p-value < 0.2 in this test, as well as variables that have been emphasized by literature to be important for weight or blood lipid profile were sequentially entered in to the multiple logistic regression models according to their hypothesized strengths of association with obesity, underweight or dyslipidemia. For example, for obesity and underweight, the potential confounders tested included youth age, sex, youth opioid use status, smoking status, father’s BMI, WSI, education years and smoking, as well as mother’s BMI, education years and smoking. Among these factors, youth opioid use and smoking status and mother’s smoking and education status displayed a high p-value and were not included in the final models. Adjusted model 1 included basic sociodemographic characteristics (age, sex) of youth as well as parents’ BMI considered to be the most strongly related to offspring weight status and to confound for the inherited aspects of offspring overweight/underweight, or father and mother triglyceride, HDL and Cholesterol level for logistic analysis of youth hypertriglyceridemia, low HDL and hypercholesterolemia, respectively. Model2 further adjusted for fathers’ smoking status due to the previously published literature relating offspring weight to parental smoke exposure^21,22^. Adjusted model three included all variables in model 2 and additionally included father’s WSI as the indicator of the socioeconomic status of the family in which the youth grew up. Additionally, we included the results of another model in which the WSI parameter in adjusted model 3 was replaces with the number of education years of father and mother (supplemental). In all models, variables of age, education years, BMI, parental triglycerides, cholesterol and HDL were entered continuously. Furthermore, the data were analyzed by parents’ route of administration of opium. Since the majority of fathers who used opium regularly reported inhaling as the route of consumption (95.1%, Table 2), we performed multiple logistic analysis in which the association of fathers’ opium inhaling with young offspring’s weight status was analyzed using similar adjusted models as described above. In this analysis, data of youth whose fathers reported opium use by route of inhaling as well as youth with parents who did not consume opioids were entered in to the analyzes. Due to the statistical inadequacy of the number of mothers who met our definition criteria for smoker or regular opioid use, data for maternal smoking status and opioid use were not included in the analysis.

In order to determine the direction and magnitude of the misreporting bias, we conducted a simple quantitative bias analysis to compare our analysis results with that of the conventional result^24^. This test was performed due to the fact that opioid use is a stigmatized and sensitive behavior which may result in misreporting or recall bias leading to some degree of non-differential misclassification in this study.

Since resources to perform an internal validation study are not available for RCS yet, previously-published validation studies which are applicable to the obtained data should be used to determine the bias parameters. Using the results of an external validation study^23^ in addition to speculating based on existing conditions^18^, we estimated parameters and performed simple bias analysis. This analysis aims to correct the errors in exposure measurement (quantitative bias analysis)^24^.

All analyses were conducted in Stata V.14. All *p*-values are two-sided, and *p*-values < 0.05 and 95% confidence intervals were considered as statistically significant. The linear and quadratic trends in the odds ratios were tested using orthogonal polynomial contrasts. All analyses were conducted in Stata V.14. All p-values are two-sided, and p-values < 0.05 and 95% confidence intervals not including 1 were considered as statistically significant.

## Results

A total of 840 parent–child trios were identified, accounting for 28% (840/3000) of the total youth participants enrolled in YRCS, and 16.8% of adult RCS (1680/10,000). Normal weight, underweight, overweight and obese youth comprised 51.19, 16.67, 19.88 and 12.26 percentages of the studied population, respectively.

Table 1 depicts the data for offspring BMI with age, sex, opioid use and smoking characteristics of youth objects, and the parental characteristics i.e. age, education years, BMI, smoking and opioid use. Among the 840 young participants (43.6% female), the rate of maternal opioid use was relatively low (1.1%). However, as Table 1 represents, a high proportion was observed for the paternal regular opioid use (42.8%), which is concordant with the previously reported high prevalence of regular opioid use among men in RCS^18^. From fathers exposed to opioid regularly, 43% started this habit before birth of the young offspring (155/360). The median value for the duration of paternal opioid use was 7 and 12.5 years pre or post conception, respectively. The majority of fathers who used opioids regularly, reported opium as substance type (98.3%) (Table 1) and inhaling as the route of consumption (95.1%) (Table 2).

**Table 1:** Baseline characteristics of the study population categorized by BMI.

Youth	≥ 18 (underweight)	< 18	p-value	≤ 25	> 25 (overweight/obesity)	p-value	Total
**Sex (%)**			0.025^b^			0.020^b^	
Female	293 (80)	73 (20)		264 (72.1)	102 (27.9)		366 (43.6)
Male	407 (85.9)	67 (14.1)		306 (64.6)	168 (35.4)		474 (56.4)
**Regular opioid use (%)**			0.604^a^			0.988^b^	
No	678 (83.2)	137 (16.8)		553 (67.8)	262 (32.2)		815 (97)
Yes	22 (88)	3 (12)		17 (68)	8 (32)		25 (3)
**Smoking (%)**			0.153^a^			0.748^b^	
No	666 (82.9)	137 (17.1)		544 (67.8)	259 (32.2)		803 (95.6)
Yes	34 (91.9)	3 (8.1)		26 (70.3)	11 (29.7)		37 (4.4)
**Father smoking (%)**			0.064^b^			0.052^b^	
No	308 (85.6)	52 (14.4)		237 (65.8)	123 (34.2)		360 (42.8)
Before	351 (82.8)	73 (17.2)		287 (67.7)	137 (32.3)		424 (50.5)
After	41 (73.2)	15 (26.8)		46 (82.1)	10 (17.9)		56 (6.7)
**Father regular opioid use (%)**			0.650^b^			0.677^b^	
No	398 (82.9)	82 (17.1)		330 (68.8)	150 (31.2)		480 (57.1)
Before	133 (85.8)	22 (14.2)		106 (68.4)	49 (31.6)		155 (18.5)
After	169 (82.4)	36 (17.6)		134 (65.4)	71 (34.6)		205 (24.4)
**Father obesity (%)**			0.000^b^			0.000^b^	
No	269 (77.3)	79 (22.7)		271 (77.8)	77 (22.2)		348 (41.4)
Yes	431 (87.6)	61 (12.4)		299 (60.8)	193 (39.2)		492 (58.6)
**Mother regular opioid use (%)**			1^a^			0.932^b^	
No	647 (83.2)	131 (16.8)		529 (68)	249 (32)		778 (98.9)
Yes	7 (77.8)	2 (22.2)		6 (66.7)	3 (33.3)		9 (1.1)
**Mother smoking (%)**			0.240^a^			0.284^a^	
No	640 (82.8)	133 (17.2)		528 (68.3)	245 (31.7)		773 (98.2)
Before	11 (100)	0 (0)		6 (54.6)	5 (45.4)		11 (1.4)
After	3 (100)	0 (0)		1 (33.3)	2 (66.7)		3 (0.4)
**Mother obesity (%)**			0.008^b^			0.000^b^	
No	339 (80)	85 (20)		331 (78.1)	93 (21.9)		424 (53.9)
Yes	315 (87)	47 (13)		203 (56.1)	159 (43.9)		362 (46.1)
**Drug type**						0.040^a^	
Opium	394 (84.4)	73 (15.6)		313 (67)	154 (33)		467 (98.3)
Heroin	3 (100)	0 (0)		0 (0)	3 (100)		3 (0.6)
Shireh	3 (60)	2 (40)		4 (80)	1 (20)		5 (1.1)
**Mean ± SD**							
Age	21.74 ± 5.00	18.95 ± 3.74	0.000	20.86 ± 4.80	22.14 ± 5.07	0.000	
Father education years	9.27 ± 4.60	9.31 ± 4.17	0.921	9.1 ± 4.39	9.65 ± 4.79	0.094	
Mother education years	8.11 ± 4.27	8.71 ± 4.09	0.137	8.20 ± 4.25	8.23 ± 4.23	0.920	
Mother BMI	29.92 ± 5.46	27.85 ± 4.24	0.000	28.57 ± 4.52	31.68 ± 6.24	0.000	
Father BMI	26.33 ± 4.29	23.86 ± 3.97	0.000	25.21 ± 4.20	27.41 ± 4.25	0.000	
WSI father	0.12 ± 0.83	0.30 ± 0.83	0.0049	0.19 ± 0.83	0.13 ± 0.814	0.4356	

Data are given as mean ± SD or absolute number n (percentage).

p-values for differences between categories were obtained using the t-test for continuous variables and using the χ^2^ or Fisher Exact Test for categorical variables.

BMI: body mass index.

^a^Fisher's exact test.

^b^Chi square test.

**Table 2:** Opioid consumption characteristics of the study population.

	Non user	Before child birth	After child birth	Total	p-value
**Opioid father route of administration**					0.018^b^
Oral	0 (0)	11 (47.8)	12 (52.2)	23 (4.9)	
Inhaling	115 (25.4)	144 (31.9)	193 (42.7)	452 (95.1)	
**Regular opioid use**					0.000^a^
Father	455 (58.5)	142 (18.2)	181 (23.3)		
Mother	0 (0)	1 (11.1)	8 (88.9)		

Data are given as absolute number n (percentage).

BMI: body mass index.

^a^Fisher's Exact Test.

^b^χ^2^ test.

### BMI

Table 3 represents the results of the multiple logistic regression models estimating the crude and adjusted association of paternal opioid use with youth BMI status. For the regression analysis on underweight, youth were divided to two categories: underweight compared to all others (non-underweight category); and for regression estimation on overweight/obesity the categories were: overweight/obese and all others (not overweight/obese category).

**Table 3:** Estimated crude and adjusted odds ratios for the overweight/obesity, underweight, high Tg, high Chol. and low HDL in Offspring, as predicted by paternal exposure to opioid.

	Crude model		Adjusted model 1		Adjusted model 2		Adjusted model 3	
	OR (95% CI)	p-value	OR (95% CI)	p-value	OR (95% CI)	p-value	OR (95% CI)	p-value
**Father regular opioid use (all routs of administration)**								
Overweight/obesity**								
Before child birth	1.01 (0.68–1.50)	0.933	1.28 (0.82–1.99)	0.272^a^	1.48 (0.92–2.36)	0.101^b^	1.51 (0.94–2.42)	0.084^c^
After child birth	1.16 (0.82–1.64)	0.386	1.52 (1.01–2.27)	0.041^a^	1.72 (1.12–2.65)	0.012^b^	1.76 (1.15–2.71)	0.009^c^
Underweight*								
Before child birth	0.80 (0.48–1.33)	0.399	0.56 (0.31–1.00)	0.050^a^	0.45 (0.24–0.84)	0.012^b^	0.46 (0.25–0.86)	0.015^c^
After child birth	1.03 (0.67–1.59)	0.880	0.79 (0.48–1.30)	0.362^a^	0.65 (0.38–1.11)	0.121^b^	0.66 (0.38–1.13)	0.134^c^
Tg > 150								
Before child birth	1.27 (0.83–1.93)	0.261	1.32 (0.83–2.08)	0.227^d^	1.32 (0.82–2.15)	0.245^e^	1.28 (0.79–2.09)	0.302f.
After child birth	1.40 (0.96–2.05)	0.073	1.36 (0.90–2.06)	0.137^d^	1.37 (0.88–2.11)	0.155^e^	1.33 (0.86–2.06)	0.194f.
Chol > 200								
Before child birth	1.03 (0.54–1.95)	0.914	1.01 (0.51–2.03)	0.958^ g^	0.90 (0.44–1.84)	0.780^ h^	0.89 (0.43–1.83)	0.755^i^
After child birth	1.44 (0.85–2.44)	0.166	1.31 (0.74–2.32	0.346^ g^	1.16 (0.64–2.13)	0.609^ h^	1.15 (0.63–2.11)	0.634^i^
HDL < 40								
Before child birth	1.25 (0.81–1.94)	0.301	1.22 (0.73–2.05)	0.438^j^	1.36 (0.78–2.37)	0.273^ k^	1.34 (0.76–2.33)	0.302^ l^
After child birth	0.85 (0.55–1.31)	0.479	0.84 (0.51–2.05)	0.506^j^	0.92 (0.54–1.57)	0.775^ k^	0.91 (0.53–1.55)	0.744^ l^
**Father regular opioid inhaling**								
Overweight/obesity**								
Before child birth	1.06 (0.71–1.58)	0.753	1.34 (0.85–2.11)	0.197^a^	1.56 (0.96–2.53)	0.066^b^	1.60 (0.99–2.59)	0.055^c^
After child birth	1.19 (0.84–1.70)	0.318	1.47 (0.98–2.23)	0.061^a^	1.69 (1.09–2.61)	0.017^b^	1.73 (1.12–2.68)	0.014^c^
Underweight*								
Before child birth	0.73 (0.43–1.26)	0.268	0.49 (0.26–0.91)	0.026^a^	0.40 (0.21–0.77)	0.006^b^	0.41 (0.21–0.78)	0.007^c^
After child birth	1.11 (0.72–1.71)	0.628	0.89 (0.54–1.48)	0.675^a^	0.74 (0.43–1.27)	0.277^b^	0.74 (0.43–1.28)	0.286^c^
**Father regular opioid inhaling** **Sensitivity analysis (by young adult sex)**								
Sons								
Overweight/obesity**								
Before child birth	1.36 (0.82–2.27)	0.227	1.83 (1.01–3.31)	0.044^ m^	2.27 (1.20–4.31)	0.012^n^	2.41 (1.26–4.58)	0.007^o^
After child birth	1.80 (1.14–2.83)	0.011	2.36 (1.38–4.02)	0.002^ m^	2.85 (1.60–5.06)	0.000^n^	3.01 (1.68–5.39)	0.000^o^
Underweight*								
Before child birth	0.84 (0.40–1.79)	0.666	0.55 (0.23–1.33)	0.191^ m^	0.40 (0.15–1.04)	0.062^n^	0.40 (0.15–1.04)	0.061^o^
After child birth	1.29 (0.70–2.38)	0.397	1.47 (0.72–2.98)	0.285^ m^	1.11 (0.51–2.40)	0.776^n^	1.11 (0.51–2.39)	0.781^o^
Daughters								
Overweight/obesity**								
Before child birth	0.70 (0.36–1.36)	0.299	0.93 (0.45–1.94)	0.862^ m^	1.02 (0.47–2.19)	0.948^n^	1.02 (0.47–2.19)	0.955^o^
After child birth	0.59 (0.32–1.10)	0.099	0.68 (0.34–1.38)	0.290^ m^	0.74 (0.36–1.55)	0.436^n^	0.74 (0.35–1.54)	0.431^o^
Underweight*								
Before child birth	0.66 (0.30–1.44)	0.299	0.46 (0.19–1.10)	0.083^ m^	0.39 (0.15–0.98)	0.046^n^	0.43 (0.17–1.10)	0.081^o^
After child birth	0.97 (0.52–1.82)	0.939	0.61 (0.29–1.27)	0.191^ m^	0.52 (0.24–1.15)	0.108^n^	0.53 (0.24–1.17)	0.117^o^

OR odds ratio, CI confidence interval, BMI: body mass index, HDL: high density lipoprotein, Chol: cholesterol. Serum HDL-Chol, WSI: socioeconomic index.

^a^Adjusted for age, sex, father and mother BMI.

^b^Adjusted for age, sex, father and mother BMI, father smoking.

^c^Adjusted for age, sex, father and mother BMI, father smoking, father WSI.

^d^Adjusted for age, sex, father and mother Tg.

^e^Adjusted for age, sex, father and mother Tg, father smoking.

^f^Adjusted for age, sex, father and mother Tg, father smoking, father WSI.

^g^Adjusted for age, sex, father and mother Chol.

^h^Adjusted for age, sex, father and mother Chol, father smoking.

^i^Adjusted for age, sex, father and mother Chol, father smoking, father WSI.

^j^Adjusted for age, sex, father and mother HDL.

^k^Adjusted for age, sex, father and mother HDL, father smoking.

^l^Adjusted for age, sex, father and mother HDL, Father smoking, father WSI.

^m^Adjusted for age, father and mother BMI.

^n^Adjusted for age, father and mother BMI, father smoking.

^o^Adjusted for age, father and mother BMI, father smoking, father WSI.

*Underweight: BMI < 18.

**Overweight/obesity: BMI > 25.

In the crude regression model, the odds of obesity (OR 1.16 (95% CI 0.82–1.64)) and underweight (OR 1.03 (95% CI 0.67–1.59)) in offspring of fathers who started using opioid regularly after child birth compared with non-users did not show a statistically significant association. However, adjusting for age, sex of youth and BMI of parents in adjusted model1, the results displayed that paternal regular opioid use started after child birth is associated with 52% increased odds ratio (OR) of overweight/obesity (adjusted OR 1.52 (95% CI 1.01–2.27)). In adjusted model2 the smoking status of fathers was added to the confounders which was shown by other publications to affect offspring metabolism, and in our bivariate logistic regressions displayed significant association with 72% increased risk of offspring obesity (adjusted OR 1.72 (95% CI 1.12–2.65)). In the third adjusted model, a parameter of fathers’ socioeconomic status named WSI (representing family’s socioeconomic state) was added to the factors in model 2. In this model, again the association persisted between paternal regular opioid use started after child birth and the young offspring’s higher odds ratio of obesity (1.76 (95% CI 1.15–2.71).

Albeit nonsignificant, paternal opioid use started from before child conception, was associated with 51% increased odds ratio for overweight/obesity (adjusted OR 1.51 (95% CI 0.94–2.42) in adjusted model 3. Moreover, paternal opioid consumption pre-fatherhood was significantly associated with 54% lowered risk of underweight (adjusted OR 0.46 (95% CI 0.25–0.86)).

The majority of fathers who used opioids regularly, reported route of inhaling (95.1%) (Table 2). Therefore, we performed multiple regression analysis, with the same models described above to estimate the odds ratio of obesity or underweight in young offspring of fathers who regularly inhaled opioid in comparison to fathers who did not use opioids. Similar to the results for general data on regular opioid use by fathers, the crude model did not show a statistically significant association (OR 1.19 (95% CI 0.84–1.70). Adjusting for confounders in adjusted models, the results of adjusted model 1(OR 1.47 (95% CI 0.98–2.23)), adjusted model 2 (OR 1.69 (95% CI 1.09–2.61)) and adjusted model 3 (OR 1.73 (95% CI 1.12–2.68) displayed a significant association between paternal regular opioid use started after child birth with higher odds ratio of obesity in young offspring. In regard to the type of the opioid used, the data indicates that the majority of participants who reported regular use of opium, and only a small minority reported heroin (0.6%) and derivatives of opium, sookhteh and shireh (1.1%) (Table 1). Therefore, in our analysis we did not perform multiple regression analysis categorizing based on the opioid type.

Next, we performed a sensitivity analysis in sons and daughters, since some of the increased odds of obesity may be due to the probable residual confounding effect of sex on exposure to paternal opioid inhaling. We found that, paternal regular use of opioid (inhaling) started after child birth was associated with significant increased odds ratio of obesity (201% in final model) in young male offspring indicated by the results of adjusted models 1 to 3 (model 1: adjusted OR 2.36 (95% CI 1.38–4.02)); model 2: adjusted OR 2.85 (95% CI 1.60–5.06) and model 3: adjusted OR 3.01 (95% CI 1.68–5.39). However, in daughters, the data does not support a significant association between obesity and paternal regular opioid inhaling started after child birth in the three adjusted models (Model 1: adjusted OR 0.68 (95% CI 0.34–1.38), model 2: adjusted OR 0.74 (95% CI 0.36–1.55) and model 3: adjusted OR 0.74 (95% CI 0.35–1.54)).

Next, we evaluated whether there is a dose-wise effect for the duration of paternal opioid use and the abovementioned phenotypes in youth. The values for the number of years of paternal opioid use after child birth for the 25th, 50th and 75th percentiles of the individuals whose fathers regularly used opioids, are 7, 12.5 and 18.5 years, respectively. A dose–response effect was observed between the number of years of paternal opioid use started after child birth (categorized based on abovementioned percentiles) and the risk of overweight/obesity. 1 to 7 years of paternal opioid use after child birth was associated with 72% higher risk of overweight/obesity (adjusted OR 1.72 (95% CI 0.96–3.05)), 8–12.5 years with 119% (adjusted OR 2.19 (95% CI 1.20–4.00)), and 13 to 18.5 years with 190% (adjusted OR 2.90 (95% CI 1.60–5.25)). Orthogonal polynomial contrast test indicated a linear correlation between the odds ratios of 25th, 50th and 75th percentiles (p-trend = 0.0008). However, more than 18.5 years of paternal opioid use after child birth did not follow this linear correlation (adjusted OR1.10 (95% CI 0.60–1.99)) (Table 4), and including this category in polynomial analysis suggests a quadratic trend between odds ratios (p-trend = 0.0011). This result further suggests that the environmental impacts of paternal regular opioid use are related to offspring’s increased risk of obesity in a dose-responsive manner.

**Table 4:** Estimated crude and adjusted odds ratios for the overweight/obesity and underweight, as predicted by duration of paternal exposure to opioid.

	Crude model		Final adjusted model	
	OR (95% CI)	p-value	OR (95% CI)	p-value
**Father regular opioid use (all routes of consumption)**^**a**^				
**Duration of paternal opioid use post-conception**^**b**^				
Overweight/obesity				p-trend = 0.0008^#^
1–7 years	0.90 (0.54–1.47)	0.676	1.72 (0.96–3.05)	0.064
8–12.5 years	1.50 (0.93–2.42)	0.091	2.19 (1.20–4.00)	0.010
13–18.5 years	1.10 (0.68–1.79)	0.679	2.90 (1.60–5.25)	0.000
18.5 < years	1.05 (0.64–1.71)	0.840	1.10 (0.60–1.99)	0.751
**Duration of paternal opioid use before conception**^**c**^				
Underweight				
1–4 years	0.80 (0.32–1.94)	0.624	0.42 (0.14–1.30)	0.135
5–7 years	0.58 (0.20–1.67)	0.316	0.40 (0.12–1.28)	0.124
7–12 years	1.31 (0.61–2.81)	0.487	1.03 (0.45–2.33)	0.939
12 < years	0.46 (0.13–1.54)	0.212	0.30 (0.06–1.37)	0.123

OR odds ratio, CI confidence interval, BMI: body mass index.

^a,b,c^Adjusted for age, sex, father and mother, BMI, paternal smoking, and father and mother education years.

*Underweight: BMI < 18.

**Overweight/obesity: BMI > 25.

^#^pTrend of linear correlation is estimated for values of duration of paternal opioid use post-conception which are lower than 19 years.

In order to obtain further insight on the potential pre-conceptional (transgenerational) effects of paternal opioid use, we tested the relationship between the duration of paternal opioid use before child birth with the risk of underweight. The values for the number of years of paternal opioid use before child birth in 25th, 50th and 75th percentiles of the individuals with opioid user fathers, are 4, 7 and 12 years, respectively, which were used to categorize the population. Based on the post-estimation orthogonal polynomial contrast, no linear or quadratic trend of significantly increasing levels of association was observed in this analysis.

Finally, the direction and magnitude of the non-differential misreporting bias was determined. For this purpose, we compared the results of quantitative bias analysis and the conventional results when the specificity and sensitivity of the self-reported opioid use was assumed to be 90% among parents of the current study (see supplemental Table 4). The adjusted odds ratio for this bias about obesity in relation to the status of paternal opioid use after child birth is 1.24. These Odds ratios were reported in the crude logistics model before adjusting for this bias as 1.16. Based on this analysis (on condition of the accuracy of the values assigned to the bias parameters), percent biases, were − 6%, indicating that the odds ratio increased or, on the other words, there was 6% error towards null before adjusting for this bias (supplemental Table 4).

### Lipid profile

As depicted in Table 3, our regression analysis showed that paternal regular opioid use started either pre or post child conception, did not display a significant association with odds ratio of high plasma levels of triglyceride (TG > 150), hypercholesterolemia (cholesterol > 200) or low HDL (Table 3).

Overall, our analyses support a significant post-conceptional relationship between paternal opioid use and offspring’s risk of obesity, emphasizing on the environmental impact of paternal opioid consumption on offspring weight. However, a clear conclusion on the potential transgenerational impact of paternal opioid use on offspring weight is not possible based on the present results; and future studies with larger population sizes are required.

## Discussion

The present study is conducted on participants of RCS^18^, as one of the district areas of the prospective PERSIAN cohort^17,25^. Multiple logistic regression analyses were undertaken in 840 trios of parent–child assessing the association of paternal opioid consumption with offspring BMI and the plasma lipid components (triglyceride, cholesterol and HDL). Despite the high rate of substance use among parents and its burden on public health care, epidemiological studies have mainly focused on the offspring neonatal withdrawal syndrome and short-term effects in the offspring, or are mainly limited to the psychological and neurological symptoms^12,15,26–28^. However, evidences so far support the notion that parental substance use (cocaine and alcohol) may exert long effects on offspring metabolism modifying the risk of adult-onset disease such as diabetes^9^. To the best of our knowledge, this is the first ever study who checked paternal-line opioid relationship with offspring metabolic indices BMI and plasma lipid components.

In the current study, we found a significant dose-sensitive increase in overweight/obesity odds ratio associated with paternal years of opioid use started after child birth. The effect persisted when subjects that regularly used opioids by non-inhaling routes were excluded from our analysis. This result indicates that even without any genetic or epigenetic transgenerational influences, the environmental impacts of paternal opioid use may be sufficient to alter offspring metabolism. Our sensitivity analysis in sons and daughters indicates that paternal regular use of opioid (inhaling) is associated with higher odds ratio of obesity only in sons (201% increase in final adjusted model), and no significant increase was observed in daughters. The underlying reason for this differential response of male and female offspring to parental opioid inhaling may be a cultural factor that sons are more in company of their fathers at the time of opioid inhaling, or it may be the biologically known sex differences in response to opioids based on the differences in neuroimmune and hormonal systems^29–34^.

On the other hand, we found that paternal opioid use pre-fatherhood is significantly associated lower odds ratio of underweight in offspring. However, our results did not statistically support a dose-responsive relationship between the duration of paternal opioid use before child birth with offspring BMI status. Given that the majority of fathers who use opioid started the habit after child birth (57%), it is possible that the size of the sample for pre-fatherhood cases is not large enough to support adequate statistical power to detect an association with overweight/obesity or dose–response effects on underweight. However, this could also be due to a lack of association when environmental effects of paternal opioid use are combined with the potential transgenerational impacts? Future studies are required to answer this question, studying larger populations.

Animal studies carried out by Najafipour et al. showed that a long term passive opium smoke exposure results in slightly higher cholesterol and LDL-cholesterol levels in rabbits^14^. Here, we did not observe a significant association between paternal opioid consumption and offspring dyslipidemia. Future population-based and experimental analysis with larger populations are required to provide higher statistical power in order to investigate whether plasma lipid profile may be affected by exposure to second-hand opioid smoke or via transgenerational transfer of effects of paternal or maternal opioid consumption preconception. To further investigate the environmental impacts of paternal opioid consumption, we propose future prospective studies to assess the BMI status and blood biochemical parameters of the female partners (mothers) in the studied trios, to investigate the potential environmental impact of husband’s opioid use on partner’s metabolic indices.

Due to the low percentage of maternal regular opioid use, we did not include analysis regarding the relationship of maternal use and offspring weight and lipid profile. Future prospective studies on the effects of maternal opioid use specifically during pregnancy and/or post-partum and lactation periods are warranted to investigate the offspring’s risk of developing late-onset non-communicable diseases (NCDs) such as obesity, cardiometabolic complications, dyslipidemia and diabetes. Currently a prospective birth cohort project is ongoing (at the recruitment stage) in RCS (profile not published yet) which may provide valuable data regarding this question in the future.

A strength of the present study is that the data on blood chemistry and anthropometry is not based on self-reports; and complete physical examination for anthropometric measurements, and blood analysis by Rafsanjan Cohort Center specialized pathology laboratory, provides direct data on biochemical metabolic parameters and BMI^18^. In addition, further information including medical and family history of participants are collected via comprehensive computer-assisted, server-based questionnaires completed by trained experts according to the PERSIAN cohort protocols^17^.

Limitations of the current study include the size of the sample (840trios) which may have affected the power of analysis. Since opioid use rates are relatively high in our cit^18^, we propose future studies that assess the relationship of parental opioid inhaling and young and adult offspring’s metabolic health status recruiting larger number of parent–offspring trios in this geographic region.

Another limitation of our study is the fact that information on drug use is based on self-reports of individuals participating in the interviews. Therefore, some participants may have not reported their opioid use or misinformed the interviewer about the type of the drug they use or the route of administration. On the other hand, some degree of misclassification due to self-reporting and recall biases is possible. Different studies aimed to evaluate the bias in opioid use self-reporting have tested biological samples for residues of opioid, and have shown that opioid use data collected based on self-reporting may lead to misreporting^35–39^. Thus, measurement errors such as self-reporting bias may arise leading to some deviations from reality (incidence of bias in estimates). Nevertheless, the level of this self-reporting bias is dependent on sex, age, type of substance, socio-cultural and geographical characteristics of the study population^37,40,41^. Rate of self-reporting opioid consumption in the current study population is relatively high^18^, probably due to its lower associated social stigma. Knowing this fact, makes us believe that the validity of our data on opioid use in RCS population is probably relatively high. In a comparable study by Abnet et al. a high rate of sensitivity of opium use was reported in a Turkmen population in Iran which are known to use opioid as a traditional medicine and this habit is associated with low social stigma among^23^.

Although the results of our simple bias analysis indicate that the direction and magnitude of this bias is probably towards null and the adjusted odds ratio for this bias about obesity is stronger than that of the conventional result, the accuracy of the results of this model is robustly affected by the precision of the bias parameters^24^. To determine the bias parameters, we used the results of an external validation study since no internal validation study has been performed in RCS to evaluate the self-reporting of opioid use, and also deduced parameters based on existing conditions^18^. Future internal validation studies are warranted for RCS to estimate the magnitude and direction of this bias more accurately.

After Covid-19 pandemic it is noteworthy to ask whether prospective cohort studies such as RCS are going to be affected in the future due to the characterized and uncharacterized Covid-associated health consequences that the infected individuals may experience over time. How it might affect the relationship of NCDs with the known risk factors in the future? This potential impact may be investigated during the follow up of the present cohort study in the following years.

In conclusion, the results of the present study are indirectly implicative of the potential environmental impact of opioid use by fathers on obesity; and suggest that parental regular opioid use may be considered as a new risk factor for obesity in the future.

## Supplementary Information

Supplementary Information 1.

Supplementary Information 2.
