# Supplementary material for: Cohort-based analysis of paternal opioid use in relation to offspring’s BMI and plasma lipid profile
Source: Sci Rep. 2021 May 4;11:9462. doi: 10.1038/s41598-021-88781-9 (PMC8096835; doi:10.1038/s41598-021-88781-9)
Supplement: Supplementary file 1 — Supplementary Information 1. [file 41598_2021_88781_MOESM1_ESM.docx]

| **Supplemental Table 1.** Baseline characteristics of the study population categorized by serum concentration of triglyceride | | | | |
| --- | --- | --- | --- | --- |
|  | **Tg <150** | **Tg >=150** | **Total** | **p-Value** |
| **Sex (%)** |  |  |  | 0.000 |
| Female | 313 (85.52) | 53 (14.48) | 366(43.57) |  |
| Male | 327 (68.99) | 147 (31.01) | 474 (56.43) |  |
| **Addiction (%)** |  |  |  | 0.001 |
| No | 628 (77.06) | 187 (22.94) | 815 (97.02) |  |
| Yes | 12 (48.00) | 13 (52.00) | 25 (2.98) |  |
| **Smoking (%)** |  |  |  | 0.040 |
| No | 617 (76.84) | 186 (23.16) | 803 (95.60) |  |
| Yes | 23 (62.16) | 14 (37.84) | 37 (4.40) |  |
| **father Smoking (%)** |  |  |  | 0.804 |
| No | 278 (77.22) | 82 (22.78) | 360 (42.86) |  |
| Before | 319 (75.24) | 105 (24.76) | 424 (50.48) |  |
| After | 43 (76.79) | 13 (23.21) | 56 (6.67) |  |
| **father opioid user (%)** |  |  |  | 0.165 |
| No | 377 (78.54) | 103 (21.46) | 480 (57.14) |  |
| Before | 115 (74.19) | 40 (25.81) | 155 (18.45) |  |
| After | 148 (72.20) | 57 (27.80) | 205 (24.40) |  |
| **Mother Smoking (%)** |  |  |  | 0.913 |
| No | 585 (75.68) | 188 (24.32) | 773 (98.22) |  |
| Before | 8 (72.73) | 3 (27.27) | 11 (1.40) |  |
| After | 2 (66.67) | 1 (33.33) | 3 (0.38) |  |
| **Mother opioid user (%)** |  |  |  | 0.159 |
| No | 590 (75.84) | 188 (24.16) | 778 (98.86) |  |
| Yes | 5 (55.56) | 4 (44.44) | 9 (1.14) |  |
| **Mean ± SD** |  |  |  |  |
| Age | 20.78±4.72 | 22.85±5.26 |  | 0.000 |
| Father education years | 9.46±4.54 | 8.68±4.45 |  | 0.031 |
| Mother education years | 8.50±4.20 | 7.30±4.24 |  | 0.000 |
| Mother BMI | 29.41±5.29 | 30.06±5.46 |  | 0.145 |
| Father BMI | 25.89±4.25 | 26.01±4.62 |  | 0.733 |

Data are given as mean ± SD or absolute number n (percentage). p-values for differences between categories were obtained using the t-test for continuous variables and using the χ2 for categorical variables. BMI: body mass index, Tg: triglyceride. Tg is measured by standard laboratory tests in collected blood samples of study subjects.

| **Supplemental Table 2.**  Baseline characteristics of the study population categorized by serum concentration of cholesterol | | | | |
| --- | --- | --- | --- | --- |
|  | **Chol <=200** | **Chol >200** | **Total** | **p-Value** |
| **Sex (%)** |  |  |  | 0.761 |
| Female | 332 (90.71) | 34 (9.29) | 366 (43.57) |  |
| Male | 427 (90.08) | 47 (9.92) | 474 (56.43) |  |
| **Addiction (%)** |  |  |  | 0.014 |
| No | 740 (90.80) | 75 (9.20) | 815 (97.02) |  |
| Yes | 19 (76.00) | 6 (24.00) | 25 (2.98) |  |
| **Smoking (%)** |  |  |  | 0.806 |
| No | 726 (90.41) | 77 (9.59) | 803 (95.6) |  |
| Yes | 33 (89.19) | 4 (10.81) | 37 (4.4) |  |
| **Total father Smoking (%)** |  |  |  | 0.402 |
| No | 331 (91.94) | 29 (8.06) | 360 (42.86) |  |
| Before | 378 (89.15) | 46 (10.85) | 424 (50.48) |  |
| After | 50 (89.29) | 6 (10.71) | 56 (6.67) |  |
| **Total father opioid consumption (%)** |  |  |  | 0.361 |
| No | 438 (91.25) | 42 (8.75) | 480 (57.14) |  |
| Before | 141 (90.97) | 14 (9.03) | 155 (18.45) |  |
| After | 180 (87.80) | 25 (12.20) | 205 (24.4) |  |
| **Total mother Smoking (%)** |  |  |  | 0.107 |
| No | 702 (90.82) | 71 (9.18) | 773 (98.22) |  |
| Before | 8 (72.73) | 3 (27.27) | 11 (1.4) |  |
| After | 3 (100) | 0 (0.00) | 3 (0.38) |  |
| **Total mother opioid consumption (%)** |  |  |  | 0.331 |
| No | 704 (90.49) | 74 (9.51) | 778 (98.86) |  |
| Yes | 9 (100) | 0 (0.00) | 9 (1.14) |  |
| **Mean ± SD** |  |  |  |  |
| Age | 21.06±4.85 | 23.27±5.23 |  | 0.000 |
| Father education years | 9.37±4.48 | 8.41±4.89 |  | 0.072 |
| Mother education years | 8.27±4.25 | 7.60±4.10 |  | 0.195 |
| Mother BMI | 29.52±5.25 | 30.07±6.09 |  | 0.396 |
| Father BMI | 25.95±4.34 | 25.61±4.31 |  | 0.493 |

Data are given as mean ± SD or absolute number n (percentage). p-values for differences between categories were obtained using the t- test for continuous variables and using the χ2 for categorical variables. BMI: body mass index, Chol: cholesterol.

| **Supplemental Table 3.**  Baseline characteristics of the study population categorized by serum concentration of HDL-Chol | | | | |
| --- | --- | --- | --- | --- |
|  | **HDL >40** | **HDL <40** | **Total** | **p-Value** |
| **Sex (%)** |  |  |  | 0.000 |
| Female | 361 (98.63) | 5 (1.37) | 366 (43.57) |  |
| Male | 315 (66.46) | 159 (33.54) | 474 (56.43) |  |
| **Addiction (%)** |  |  |  | 0.009 |
| No | 661 (81.10) | 154 (18.90) | 815 (97.02) |  |
| Yes | 15 (60.00) | 10 (40.00) | 25 (2.98) |  |
| **Smoking (%)** |  |  |  | 0.043 |
| No | 651 (81.07) | 152 (18.93) | 803 (95.6) |  |
| Yes | 25 (67.57) | 12 (32.43) | 37 (4.4) |  |
| **father Smoking (%)** |  |  |  | 0.574 |
| No | 287 (79.72) | 73 (20.28) | 360 (42.86) |  |
| Before | 341 (80.42) | 83 (19.58) | 424 (50.48) |  |
| After | 48 (85.71) | 8 (14.29) | 56 (6.67) |  |
| **Father opioid consumption habit (%)** |  |  |  | 0.343 |
| No | 387 (80.62) | 93 (19.38) | 480 (57.14) |  |
| Before | 119 (76.77) | 36 (23.23) | 155 (18.45) |  |
| After | 170 (82.93) | 35 (17.07) | 205 (24.4) |  |
| **Total mother Smoking (%)** |  |  |  | 0.567 |
| No | 621 (80.34) | 152 (19.66) | 773 (98.22) |  |
| Before | 8 (72.73) | 3 (27.27) | 11 (1.4) |  |
| After | 3 (100) | 0 (0.00) | 3 (0.38) |  |
| **Mother opioid consumption habit (%)** |  |  |  | 0.301 |
| No | 626 (80.46) | 152 (19.54) | 778 (98.86) |  |
| Yes | 6 (66.67) | 3 (33.33) | 9 (1.14) |  |
| **Mean ± SD** |  |  |  |  |
| **Age** | 21.21±4.87 | 21.52±5.16 |  | 0.477 |
| **Father education years** | 9.16±4.50 | 9.75±4.64 |  | 0.138 |
| **Mother education years** | 8.26±4.24 | 8.03±4.24 |  | 0.548 |
| **Mother BMI** | 29.35±5.00 | 30.47±6.47 |  | 0.019 |
| **Father BMI** | 25.78±4.32 | 26.51±4.37 |  | 0.051 |

Data are given as mean ± SD or absolute number n (percentage). p-values for differences between categories were obtained using the t- test for continuous variables and using the χ2 for categorical variables. BMI: body mass index, HDL-Chol: high density lipoprotein-cholesterol.
