# Supplementary material for: Cohort-based analysis of paternal opioid use in relation to offspring’s BMI and plasma lipid profile
Source: Sci Rep. 2021 May 4;11:9462. doi: 10.1038/s41598-021-88781-9 (PMC8096835; doi:10.1038/s41598-021-88781-9)
Supplement: Supplementary file 2 — Supplementary Information 2. [file 41598_2021_88781_MOESM2_ESM.docx]

**Result for simple bias test:** The adjusted odds ratios for this bias about overweight/ obesity are 1.24. These Odds ratios were reported in the crude logistics model before adjusting for this bias as 1.17. based on this analysis (on condition of the accuracy of the values assigned to the bias parameters), percent biases, were− 6% for overweight/ obesity, indicating that the odds ratio increased or, on the other words, there was 6 % error towards null before adjusting for this bias. Under non-differential misclassification (bolded cells) bias corrected Odds ratios are always further away from the null

Supplemental table 4 . Deterministic sensitivity analysis of the **Opium use**- **overweight/obesity**

Odds ratios under various assumptions about the self-reported sensitivity (Se) and specificity (Sp) opium use among normal and **overweight/obesity**.

| normal group | | | | | **overweight/obesity** group | |
| --- | --- | --- | --- | --- | --- | --- |
| 0.8 | 0.9 | 0.8 | 0.9 | Se | Sp | Se |
| 0.8 | 0.8 | 0.9 | 0.9 | Sp |  |  |
| 2.20 | 2.63 | 1.04 | **1.24** |  | 0.9 | 0.9 |
| 2.66 | 3.18 | **1.25** | 1.50 |  | 0.9 | 0.8 |
| 1.21 | **1.44** | 0.57 | 0.68 |  | 0.8 | 0.9 |
| **1.46** | 1.74 | 0.69 | 0.82 |  | 0.8 | 0.8 |

Se: sensitivity

Sp: Specificity
